# Sources of Microstructure in Mammalian Cochlear Responses

**DOI:** 10.1007/s10162-025-00974-5

**Published:** 2025-01-29

**Authors:** James B. Dewey

**Affiliations:** Caruso Department of Otolaryngology—Head & Neck Surgery, University of Southern California, Los Angeles, CA USA

**Keywords:** Microstructure, Otoacoustic emissions, Cochlear mechanics, Wave interference

## Abstract

Quasiperiodic fluctuations with frequency are observed in a variety of responses that either originate from or strongly depend on the cochlea’s active mechanics. These spectral microstructures are unique and stable features of individual ears and have been most thoroughly studied in behavioral hearing thresholds and otoacoustic emissions (OAEs). While the exact morphology of the microstructure patterns may differ across measurement types, the patterns are interrelated and are thought to depend on common mechanisms. This review summarizes the characteristics and proposed origins of the microstructures observed in behavioral and OAE responses, as well as other mechanical and electrophysiological responses of the mammalian cochlea. Throughout, the work of Glenis Long and colleagues is highlighted. Long contributed greatly to our understanding of microstructure and its perceptual consequences, as well as to the development of techniques for reducing the impact of microstructure on OAE-based assays of cochlear function.

## Introduction

In 1958, Elliott first noted a “ripple effect” in behavioral pure-tone hearing thresholds obtained in fine frequency increments [1]. Similar quasiperiodic fluctuations with frequency—termed microstructure or fine structure—have since been observed in the amplitudes, phases, and delays of otoacoustic emissions (OAEs). Over several decades of detailed experimental work and modeling efforts, Glenis Long and colleagues characterized the microstructures evident in both behavioral and OAE responses, explored the relationships among them, and developed techniques to minimize the complicating influence that these patterns have on psychoacoustic and OAE-based investigations of cochlear function. With an emphasis on Long’s contributions, this review summarizes the basic aspects and presumed origins of microstructures observed in behavioral and OAE responses. As microstructure is generally most pronounced in humans, and psychoacoustic examinations of microstructure have almost exclusively been conducted in human subjects, the review primarily focuses on experimental findings from human ears. Nevertheless, the mechanisms underlying microstructure are thought to exist in all mammals, and relevant OAE measurements in small laboratory animals are also described. In addition, the characteristics and potential origins of microstructures observed in more invasive measures of cochlear function from such species are discussed.

### Defining and Characterizing Microstructure

All auditory responses have a gross frequency dependence that is determined by the various stages that are involved in converting acoustic energy into cochlear vibrations and neural impulses. Beyond this gross or macro-level spectral structure, however, more rapid fluctuations can often be observed when responses are obtained with sufficient frequency resolution. When distinguishable from measurement noise and repeatable over time, such fluctuations can be considered microstructure.

**Fig. 1:**
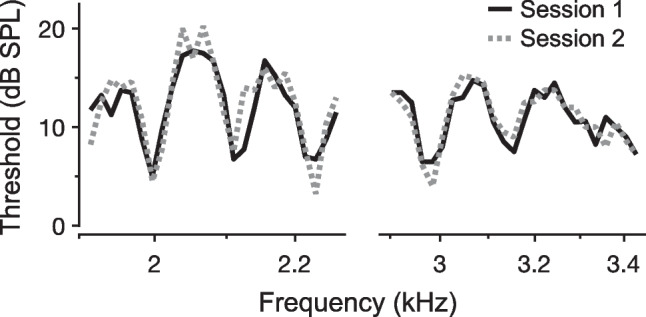
Threshold microstructure patterns and their repeatability. Pure-tone thresholds obtained in ~ 1/100th-octave steps for two 0.25-octave spans are shown for an individual human subject. For each span, solid black and dotted gray lines indicate thresholds measured in two different sessions separated by 5 days. Methods are described in [6]

For behavioral hearing thresholds and OAEs, the strength and frequency periodicity of the observed fluctuations are quite similar, and a common underlying “auditory” or “cochlear” microstructure has been assumed [2–4]. The strength or “depth” of the microstructure is quantified as the difference in magnitude between adjacent maxima and minima and can approach 20 dB. The frequency difference between adjacent maxima or minima is not constant on either linear or logarithmic frequency scales, but generally corresponds to ~ 1/10 to 1/20th of the stimulus frequency, at least for the commonly studied frequency range of ~ 1 to 4 kHz. Microstructure patterns are unique to individual ears and relatively stable over time, as demonstrated by Long through repeated measurements of her own hearing thresholds over 18 months [5]. Figure 1 shows more recent examples of threshold microstructure patterns and their repeatability over a shorter time scale in an individual ear. As described later, however, these patterns can shift in frequency and become more or less pronounced following various experimental manipulations.

### Origins of Microstructure in Psychoacoustic and OAE Measurements

Much of the microstructure observed in behavioral thresholds and OAEs can be attributed to the mechanisms that underlie so-called “reflection-source” OAEs [7]. These emissions are thought to result from coherent reflection of forward-traveling waves due to impedance irregularities that are randomly distributed along the cochlear partition [8]. At low stimulus levels, this reflection mechanism is presumed to be responsible for generating stimulus-frequency OAEs (SFOAEs) elicited by discrete- or swept-tone stimuli, as well as OAE responses to click or tone-burst stimuli (i.e., transient-evoked OAEs, or TEOAEs). The reflection mechanism is distinct from the nonlinear distortion mechanism that is the primary (or at least initial) source of distortion-product OAEs (DPOAEs) elicited by two-tone stimuli.

Microstructure is largely thought to result from constructive and destructive interference that is initiated by the reflection of forward-traveling waves within the cochlea. Such interference can result from (1) multiple reflections between the site(s) of traveling-wave reflection and the middle-ear boundary and (2) interactions between reflection- and distortion-source components. The first type of interference is thought to underlie the microstructure observed in psychophysical, SFOAE, and TEOAE measures obtained at low stimulus levels, as well as the generation of spontaneous OAEs (SOAEs). The second type is a strong source of microstructure in DPOAEs and may also contribute to the spectral structure of SFOAEs and TEOAEs at high stimulus levels. The precise origins and consequences of these different interference patterns are described below.

## Microstructure Arising from Multiple Intracochlear Reflections

The discovery of OAEs by David Kemp in the late 1970s was motivated by several puzzling and seemingly related psychoacoustic phenomena [9]. These included threshold microstructure and correlated fluctuations in loudness judgements, as well as the perception of tonal interactions like beating or distortion when presented with stimuli at frequencies near those of threshold minima [2, 10, 11].

**Fig. 2:**
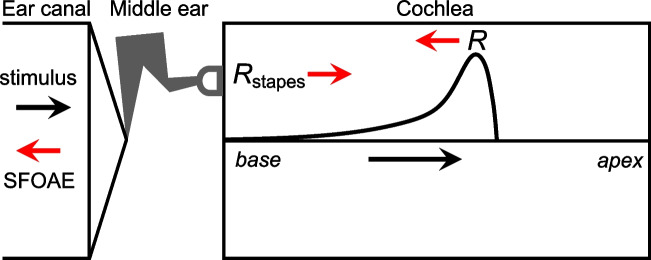
Schematic illustrating intracochlear reflections. Stimulation with a tone initiates a forward-traveling wave that is subject to scattering by micromechanical impedance irregularities, resulting in the generation of reverse-propagating waves (red, basally pointed arrow). Upon reaching the stapes, these waves vibrate the middle ear, producing an SFOAE in the ear canal. However, they are also partially re-reflected at the middle-ear boundary, initiating secondary forward-traveling waves (red, apically pointed arrow). These interfere with the stimulus-driven wave and contribute to additional rounds of reflection. Note that forward-traveling waves are reflected by spatially distributed irregularities and not a single point source. As detailed in [13], the traveling-wave reflectance, *R*, can be defined as the ratio of the net reverse-traveling wave to the forward-traveling wave, as evaluated at the stapes. Likewise, the reflectance at the middle-ear boundary, *R*_stapes_, can be defined as the ratio of reflected to incident waves at the stapes. The summed influence of multiple intracochlear reflections on the initial forward-traveling wave converges to a factor of 1/(1 – *RR*_stapes_)

As originally proposed by Kemp [2] and since re-iterated [8, 12–14], these phenomena can all be explained as the result of multiple intracochlear reflections of the waves that give rise to OAEs in the ear canal—specifically, what are now classified as reflection-source OAEs. The basic process (schematized in highly simplified form in Fig. 2) is initiated when forward-traveling waves are scattered by micromechanical impedance irregularities. Such irregularities, which are often referred to as “roughness” [12], presumably arise from variations in the structure or function of the organ of Corti along its length. For example, impedance irregularities could result from variations in the number, mechanical properties, and arrangement of the hair cells and supporting cells, or perhaps in the strength of any locally generated amplifying forces [8, 13]. Regardless of their precise origins, the resulting reverse-traveling waves propagate toward the stapes, where they are then re-reflected due to the impedance mismatch at the middle-ear boundary. This launches additional forward-traveling waves that can interfere constructively or destructively with the initial, stimulus-driven wave and contribute to further rounds of reflection.

Maximal constructive interference theoretically occurs at frequencies where the round-trip phase accumulation associated with wave travel from the stapes to the more apical reflection sites and back is a whole number of cycles [13]. Such frequencies should therefore be associated with local enhancements in sensitivity (i.e., threshold minima). Additionally, provided sufficient amplification of forward- and reverse-traveling waves, the reflections can become self-sustaining. The presence of such spontaneous oscillations would naturally explain the tonal interactions experienced at frequencies near threshold minima. Of course, the existence of reverse-traveling waves and spontaneous oscillations was demonstrated by Kemp’s measurements of both evoked and spontaneous OAEs (SOAEs) in the ear canal [15, 16], which provided firm support for this framework.

### Correlations Among Microstructures in Thresholds and Reflection-Source OAEs

**Fig. 3:**
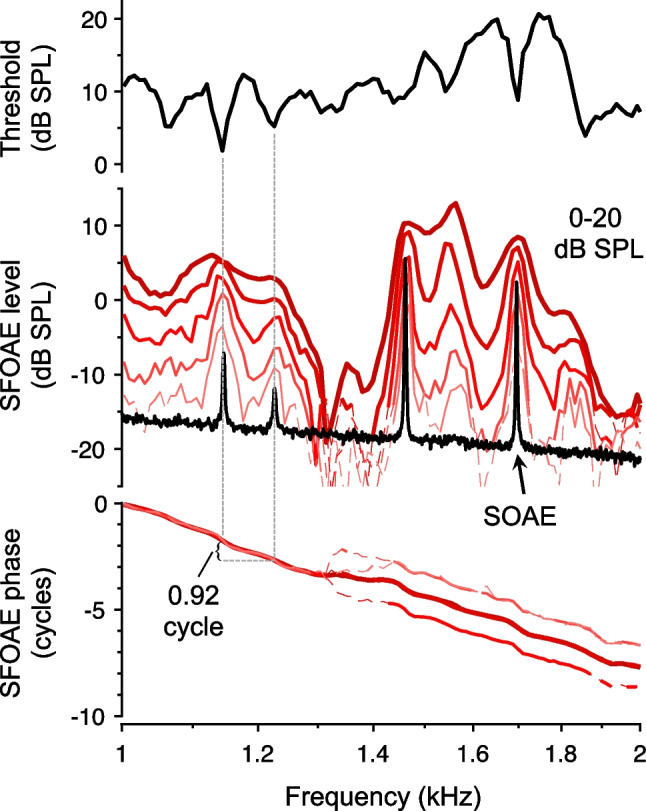
Relationships among hearing thresholds, SFOAEs, and SOAEs. Pure-tone thresholds (top), SFOAE amplitudes (middle), and SFOAE phases (bottom) measured in 1/100th-octave steps from 1 to 2 kHz are shown for an individual human subject. SFOAE responses were obtained for 0–20 dB SPL stimuli in 5 dB steps (thicker/darker lines indicate responses at higher stimulus levels). Thin, dashed portions of the SFOAE curves indicate responses with amplitudes less than 6 dB above the measurement noise floor. A representative ear-canal spectrum in quiet is also shown along with the SFOAE amplitudes, revealing multiple SOAEs. Threshold minima align well with SFOAE amplitude maxima and SOAEs, and roughly one cycle of SFOAE phase is accumulated for each microstructure period. Data were adapted from supplementary material provided in [4], and thresholds were previously published in [6]. See the aforementioned references for more detailed methods

Many, including Long and colleagues, have since replicated Kemp’s initial findings and provided further evidence that a common mechanism is responsible for the microstructure observed in thresholds and OAEs. As illustrated in Fig. 3, there is a close correspondence between frequencies of threshold minima, maxima in OAE amplitudes evoked by low-level tones and transient stimuli, and SOAEs [3, 5, 17, 18]. In fact, the precise morphology of the microstructure observed in hearing thresholds is nearly identical to that in SFOAEs elicited by low-level tones [4]. Furthermore, the minimum frequency spacing of adjacent threshold minima, evoked OAE maxima, and SOAEs also corresponds to the interval over which SFOAE phases accumulate one cycle [4, 13, 19, 20]. Again, this is consistent with maximal constructive interference occurring when the round-trip phase accumulated by waves traveling to the more apical, OAE-generating reflection sites and back to the stapes is an integer number of cycles. Note, however, that threshold minima and OAE amplitude maxima are not always accompanied by measurable SOAEs, since the latter occur only when the round-trip gain is sufficiently high. The detection of SOAEs is also potentially limited by middle-ear transmission and the noise floor of the measurement.

### Dependence on Cochlear Amplification

The strength of the OAE-generating reflectance depends on the amplification of traveling waves by outer hair cells (OHCs). The microstructure common to reflection-source OAEs and behavioral thresholds should therefore be highly sensitive to OHC activity and cochlear status. Through painstaking measurements, Long and colleagues provided evidence that this is indeed the case. First, Long showed that threshold microstructure is largest under conditions where OHC-mediated amplification is presumably strongest and/or unsuppressed [5]. Specifically, threshold microstructure is reduced in the presence of tonal or wideband masking stimuli and is mostly absent once masked thresholds are elevated above ~ 40 dB SPL. Such effects are consistent with the notion that amplification and intracochlear reflections are both suppressed by the masking stimuli. Intracochlear reflections are also expected to saturate at moderate stimulus levels, reducing their overall influence on any stimulus-driven waves.

Most compellingly, Long and Tubis [21, 22] demonstrated that threshold microstructure could be reversibly reduced after several days of consuming aspirin, a salicylate known to inhibit cochlear amplification through its effects on OHC electromotility [23]. Reductions in microstructure depth were closely associated with decreases in the amplitudes of tone- and click-evoked OAEs. Additionally, the frequencies of threshold minima, evoked OAE maxima, and SOAEs were shifted downward in frequency during aspirin consumption. Similar reversible changes were observed by Furst et al. following intense noise exposure [24]. The effects of aspirin and noise could result from a reduction in the magnitude and change in the phase of the OAE-generating reflectance, presumably due to reduced OHC-mediated amplification. For instance, a downward frequency shift in the microstructure pattern would theoretically result from increasing phase lags in the cochlear vibrations near the reflection sites. Phase lags are, in fact, observed after loss of amplification due to acoustic overstimulation or death [25].

The dependence of microstructure on OHC function has also been explored through activation of the medial olivocochlear (MOC) efferent pathway, which reduces OHC-mediated amplification [26]. Activating the MOC efferents with contralateral sound produces upward shifts in the frequencies of SOAEs, threshold minima, and SFOAE amplitude maxima [6, 20, 27, 28]. It also reduces microstructure in SFOAEs [20] and in hearing thresholds, at least when thresholds are measured at frequencies distant from SOAEs [6]. These effects are consistent with MOC activation causing a reduction in the magnitude of the OAE-generating reflectance as well as a phase lead. Indeed, MOC activation produces phase leads in both evoked OAEs [20] and basilar membrane motion [29].

Threshold microstructure and SOAEs are also primarily found in ears with normal hearing, indicating their general dependence on cochlear function [30, 31]. However, some threshold variations and SOAEs have instead been associated with pathology [32, 33]. A possible explanation for this is that damage can induce impedance irregularities that actually increase the OAE-generating reflectance, with the largest irregularities likely being near the boundary between areas with normal and impaired OHC function.

### Dependence on Middle-Ear Transmission

Microstructure is also sensitive to changes in the impedance at the middle-ear boundary. For instance, microstructure patterns and SOAEs are typically shifted upward in frequency by a few percent following changes in static ear-canal pressure or postural changes that influence the hydrostatic pressure at the oval window [2, 34–37]. Reductions in threshold microstructure depth and inversion of minima and maxima have also been observed under certain conditions [17]. Though somewhat variable, such effects could all theoretically be attributed to changes in the magnitude and phase of the stapes reflectance.

Note that middle-ear pathologies or certain experimental manipulations could both increase the reflectance at the stapes and reduce reverse middle-ear transmission. For instance, near-total reflection of reverse-propagating waves at the stapes could lead to strong intracochlear reflections while severely limiting the transmission of OAEs to the ear canal. Thus, one could potentially observe pronounced threshold microstructure in the absence of any measurable OAEs. Even under normal conditions, variability in middle-ear transmission across ears or across frequency likely complicates the relationship between microstructure depth and the overall level of SOAEs and evoked OAEs [4].

### Microstructure in Ear-Canal Pressure

For SFOAEs, it is important to distinguish between the microstructure observed in the actual OAE response versus the rippling observed in the total pressure measured in the ear canal, prior to extracting the SFOAE. This rippling is due to interference between the stimulus tone and evoked SFOAE and is reduced under conditions where the relative magnitude of the SFOAE is anticipated to become small (e.g., at high stimulus levels, or in the presence of a second, suppressor tone [3, 5, 38]). It is of course this latter fact that allows estimation of the stimulus pressure so that it can be subtracted from the total measured pressure, yielding the SFOAE.

**Fig. 4:**
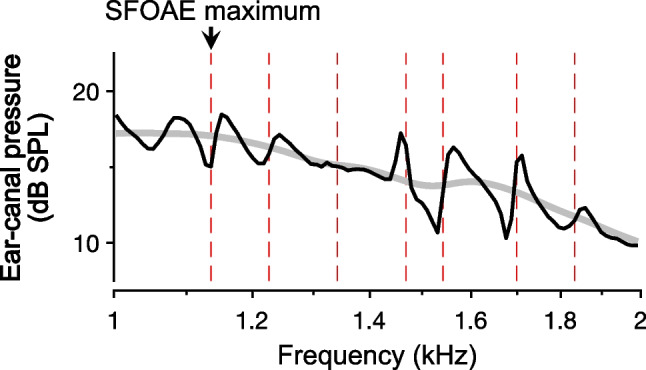
Relationship between ripples in the total ear-canal pressure and SFOAE amplitude maxima. The ear-canal pressure in response to a 15 dB SPL swept-tone stimulus (thin black curve) is compared with the response to a 60 dB SPL stimulus that has been scaled down by 45 dB (thick gray curve). Measurements are from the subject whose extracted SFOAEs are shown in Fig. 3. Ripples are present at the lower stimulus level due to interference between the stimulus and the evoked SFOAEs. However, amplitude maxima in the extracted SFOAEs (indicated by vertical dashed lines) do not align well with maxima in the ear-canal pressure. Note that the variations in stimulus pressure not attributable to interference between the stimulus and SFOAEs result from the calibration method, which attempted to achieve a constant SPL at the eardrum, rather than at the probe microphone. Relevant methods are described in [4, 6]

Like threshold and OAE microstructure, ripples in ear-canal pressure have a periodicity that is linked to the phase-vs.-frequency gradient of the SFOAE, with adjacent peaks being associated with ~ 1 cycle of SFOAE phase accumulation. However, as noted by Long, individual peaks and valleys in ear-canal pressure are not necessarily aligned well with those observed in behavioral and OAE microstructure [5] (see also [17, 39]). This is demonstrated in Fig. 4, which compares the frequencies of SFOAE amplitude maxima observed in Fig. 3 with the ear-canal pressure measured for this subject during the presentation of the 15 dB SPL stimulus. The lack of alignment is likely due to differences in the phases of the waves that are reflected at the stapes compared to the phases of those that reach the ear canal. The latter include additional delays associated with middle-ear transmission and propagation to the probe microphone [4]. Note that ripples in the ear-canal pressure would occur even if SFOAE amplitudes (and behavioral thresholds) were completely flat across frequency, exhibiting no microstructure. Such ripples only require that the SFOAE phase rotates relative to stimulus phase.

### Distinguishing Between SFOAE Micro- and Macrostructure

**Fig. 5:**
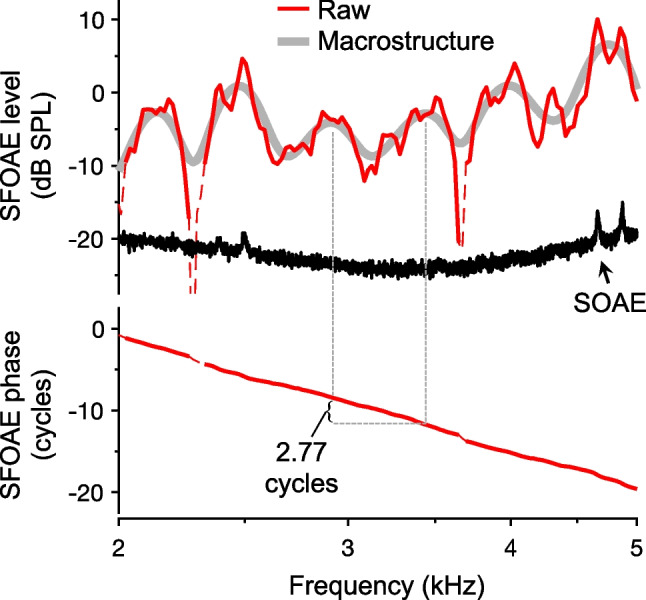
SFOAE macrostructure. Amplitudes and phases of SFOAE responses to discrete tones presented at 18 dB forward pressure level (FPL) are shown for an individual human subject. Smoothing the raw SFOAE amplitudes highlights the macrostructure, which is also quasiperiodic. The SFOAE phase accumulates roughly three cycles between adjacent macrostructure peaks. The ear-canal spectrum measured in quiet is also shown with the SFOAE amplitudes and reveals several low-level SOAEs. Thin dashed portions of the SFOAE response curves indicate responses with amplitudes less than 6 dB above the noise floor. SFOAEs were measured using the suppression method as described in [4]

It is also important to distinguish between microstructure and the peaks, valleys, and notches that instead constitute SFOAE “macrostructure” [40]. While perhaps arbitrarily defined, macrostructure is the background structure upon which the microstructure is superimposed. This background structure often appears to have a broad quasiperiodic spacing and is generally preserved across stimulus levels. While it is easier to visualize at higher stimulus levels, where the influence of microstructure is less pronounced, it can also be observed at low levels, as shown in Fig. 5. In this example, the macrostructure was approximated by smoothing the raw SFOAE amplitudes. However, since the notches that form part of the macrostructure morphology can be quite sharp, simply smoothing SFOAE spectra can remove both microstructure and macrostructure and does not always clearly separate the two.

SFOAE macrostructure was recently characterized in detail by applying a more sophisticated approach to remove the long-latency SFOAE components that are associated with multiple intracochlear reflections [40]. After effectively removing the microstructure, the distance between macrostructure peaks was found to be ~ 20–30% of the stimulus frequency. This spacing corresponds to the span over which SFOAE phase accumulates roughly three cycles (as observed in Fig. 5) and is therefore approximately three times wider than the microstructure periodicity. Though the macrostructure periodicity is apparently predicted by the coherent-reflection theory of SFOAE generation, which links this periodicity to the spatial width of the dominant reflection region (i.e., the traveling wave peak) [8, 40], its importance and interpretation remain to be more fully explored.

### Influence of SOAEs on Perception and Threshold Microstructure Morphology

SOAEs emerge from the same processes as threshold microstructure and can also directly impact microstructure morphology via interactions with the stimulus tone. Following several earlier reports [2, 3, 34, 41], Long and colleagues explored how external tones can interact with the SOAE to produce beats and sensations of “roughness” (not to be confused with the micromechanical impedance irregularities discussed earlier), or else entrain or suppress the SOAE [21, 42]. The type and strength of any interactions depend on the relative frequencies and levels of the external tone and SOAE, with suppression or entrainment occurring when the external tone becomes increasingly dominant. The boundary between perceiving a pure tone versus roughness also aligns well with that for the acoustic entrainment of an SOAE.

While SOAEs need not be associated with all threshold minima, Long demonstrated that microstructure-like patterns can be induced by presenting a low-level tone to mimic the presence of an SOAE in a frequency region lacking natural microstructure [21]. Threshold fluctuations caused by the presence of an SOAE-mimicking tone disappear when thresholds are measured using narrowband noise stimuli, which effectively eliminate perceptual cues like beating and distortion. In contrast, naturally occurring microstructure is largely preserved when using such narrowband noise stimuli. Thus, perceptual cues arising from the presence of SOAEs may “color” the threshold curve but are not required to produce pronounced microstructure.

Interestingly, the presence of an SOAE appears to result in an overall elevation of hearing thresholds at nearby frequencies. This was convincingly shown by Long and Tubis, who found that reductions in SOAE amplitudes during the early stages of aspirin consumption actually improved thresholds [22]. Threshold improvements coincident with reductions in SOAE amplitude have also been observed after noise exposure [24] and MOC activation [6]. Such improvements are probably due to a release from the neural masking that is caused by spontaneous cochlear oscillations [43]. A reduction in SOAE amplitude also changes how external stimuli interact with the SOAE [44], though whether this aids signal detection is unclear.

### Reducing the Influence of Multiple Intracochlear Reflections

Prominent microstructure can be observed in OAE responses for stimulus levels up to ~ 20 dB SPL or more, indicating that multiple intracochlear reflections significantly influence cochlear mechanics at supra-threshold levels. While studying the microstructure itself may provide insight into the mechanisms underlying intracochlear reflections, it may otherwise be desirable to minimize the influence of such reflections. Doing so provides a more straightforward window onto the form of the underlying OAE-generating reflectance and may reduce the variability in responses across ears. While this can be accomplished by simply smoothing the responses (as in Fig. 5), better results may be achieved with more advanced signal-processing techniques. These typically involve converting OAE spectra into time-domain or time–frequency-domain responses so that components with latencies indicative of later rounds of intracochlear reflection can be windowed out [8, 45, 46].

Multiple intracochlear reflections are also a significant source of inter- and intra-subject variability in perceptual studies, both at threshold and supra-threshold levels. For instance, threshold minima are associated with enhanced loudness perception for stimulus levels up to ~ 40 dB SPL [2]. Whether test stimuli are close in frequency to threshold minima or maxima also influences loudness growth [47] and the perception of amplitude modulation [48–50]. The use of tonal stimuli at moderate to high levels may reduce these effects, though this would obviously only be useful if the behavior near threshold was not of interest. Alternatively, stimuli with broader spectral energy may smooth out the variability associated with microstructure [21, 51]. This may be due to suppressive effects on intracochlear reflections and/or the stimulation of a cochlear region with characteristic frequencies that span one or more microstructure periods. However, whether or not the use of such stimuli actually reduces intra- and inter-subject variability on different psychoacoustic tasks remains to be tested. Regardless, the presence and possible influence of microstructure should be considered in psychoacoustic studies, particularly when using near-threshold stimuli.

### Alternatives to the Intracochlear Reflection Hypothesis

In mammals, the attribution of microstructure and SOAEs to multiple intracochlear reflections is by now commonplace, if not generally accepted. However, SOAEs have also been proposed to arise from individually oscillating elements (e.g., OHCs) that work either alone or through coupling with their neighbors to produce spontaneous, self-limiting activity (see [13, 52, 53] for review). Models emphasizing the role of such “local” oscillations in SOAE generation therefore contrast with those implementing the more “global” phenomenon of intracochlear wave reflection. Despite their differences, however, local oscillator models can also produce SOAEs that align with SFOAE maxima and have a frequency spacing that is associated with one cycle of SFOAE phase accumulation [54].

As discussed elsewhere [13, 52, 53], the distinction between “local” and “global” models may not be so clear, as the predictive success of local-oscillator models can depend on the degree of coupling between adjacent oscillators [55] and even on the reflection of waves at the stapes [56]. However, a key difference between these types of models remains the specific mechanism by which reverse-propagating waves are generated—i.e., traveling-wave reflection vs. intrinsic cellular oscillations. The advantage of global, wave-reflection models is perhaps that they do not require that any cochlear elements be individually capable of such oscillations, and the essential sources of wave reflection would be present regardless. Nevertheless, it is possible that aspects of both local and global frameworks co-exist in the mammalian cochlea.

It is notable that in their early modeling work, Long and colleagues replicated many of the behaviors of SOAEs, including their interactions with external stimuli, using simple van der Pol-type oscillators [21, 22, 44, 57]. However, as remarked by Shera [13], the utility of such models for approximating the response of the system as a whole does not mean that the response is actually driven by a single oscillating element. Indeed, later work from Talmadge et al. [12] embraced the intracochlear reflection framework to more comprehensively model threshold and OAE microstructures.

## Microstructure due to Interference Between Distortion and Reflection Components

**Fig. 6:**
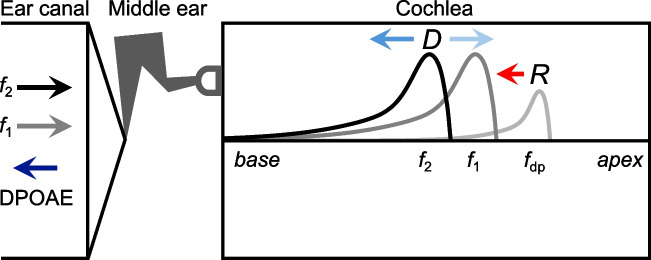
Schematic illustrating the generation of distortion and reflection components for the 2*f*_1_-*f*_2_ DPOAE. Stimulation with two tones initiates forward-traveling waves that peak at their respective tonotopic locations. Intermodulation distortion (*D*) is generated by nonlinear OHC activity in the region where the two traveling waves overlap. DPs propagating toward the base from the overlap region contribute to the primary “distortion” component of the DPOAE (blue, basally pointing arrow). However, DPs also propagate apically to the location tuned to the DP frequency (*f*_dp_). These forward-traveling DP waves are scattered by the reflection mechanism, resulting in a secondary “reflection” component (red, basally pointing arrow). Interference between the distortion and reflection components produces microstructure in the total DPOAE measured in the ear canal

Microstructure caused by the reflection mechanism is also observed in the amplitudes and phases of DPOAEs. DPOAEs are elicited with two stimulus tones at frequencies *f*_1_ and *f*_2_ (*f*_2_ > *f*_1_) and originate from the nonlinear processes involved in OHC-mediated amplification. This nonlinearity introduces mechanical intermodulation distortion products (DPs) at frequencies related to the stimuli, with the most commonly measured being 2*f*_1_-*f*_2_. For simplicity, this review primarily describes findings for the 2*f*_1_-*f*_2_ DPOAE, though the same general processes appear to influence all other DP components. As schematized in Fig. 6, 2*f*_1_-*f*_2_ DPs are generated in the region where the two stimulus-driven waves overlap, with the largest DPs typically generated near the location tuned to *f*_2_. Waves at the DP frequency then propagate away from the generation region, ultimately producing a DPOAE in the ear canal.

**Fig. 7:**
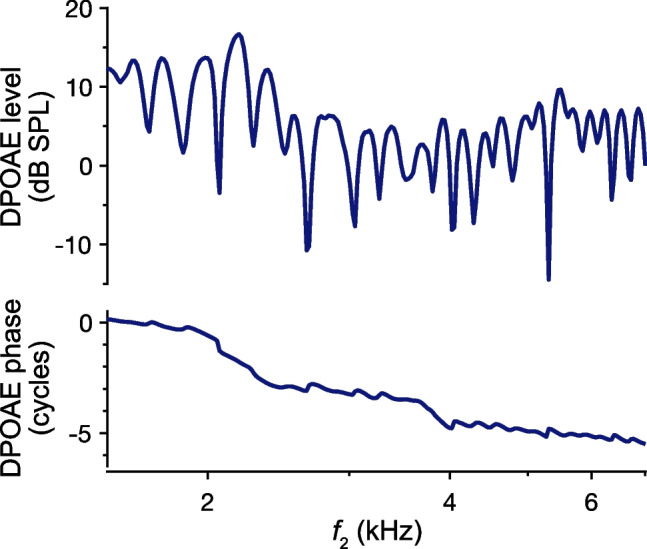
DPOAE responses in an individual human subject with strong microstructure. 2*f*_1_-*f*_2_ DPOAE amplitudes and phases are shown as a function of the *f*_2_ frequency. DPOAEs were elicited by logarithmically swept tones with an *f*_2_/*f*_1_ ratio of 1.16. The *f*_2_ and *f*_1_ tones were presented at 36 and 51 dB FPL, respectively. Methods are described in [58]

Figure 7 shows an example of 2*f*_1_-*f*_2_ DPOAE responses from an individual human subject with pronounced microstructure. This microstructure is thought to largely result from interference between reverse-propagating waves that arise via two distinct mechanisms: distortion and reflection [7] (indicated by *D* and *R*, respectively, in Fig. 6). DPs that propagate directly toward the base from the distortion-generating region near the *f*_2_ place constitute what is referred to as the primary “distortion” component. However, 2*f*_1_-*f*_2_ DPs also propagate apically to the place tuned to the DP frequency, where they are maximally amplified and scattered by the reflection mechanism. Reverse-propagating DP waves arising from this mechanism form a secondary “reflection” component, which is essentially an SFOAE elicited by the intracochlear DPs.

As the stimulus frequencies are varied, the distortion and reflection components alternate between summing constructively and destructively due to their different phase-vs.-frequency behaviors. As shown previously, reflection-source emissions are characterized by their steep phase-vs.-frequency gradients, such that they are sometimes referred to as “long-latency” components. In contrast, the distortion component has a relatively flat phase gradient when stimuli are swept at a fixed *f*_2_/*f*_1_ ratio. This is because the DP phase is tied to the phases of the stimulus-driven waves at the generation site, and these are expected to maintain a roughly constant relationship as the stimulus frequencies are varied. Since the phase gradient of the distortion component is approximately zero, this component is sometimes referred to as a zero- or short-latency component. Note that DPOAE phase gradients should not be interpreted as literal time delays, and the distortion component does not emerge instantaneously from the ear.

**Fig. 8:**
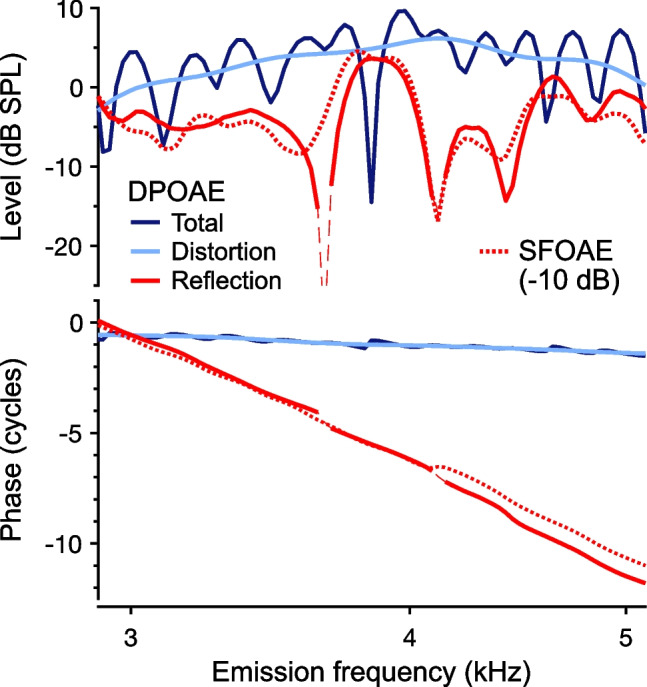
DPOAE component separation. Amplitudes and phases of the total 2*f*_1_-*f*_2_ DPOAE and separated distortion and reflection components are shown for the subject whose data are plotted in Fig. 7. Components were separated via time-domain windowing. Data are plotted vs. the emission frequency (corresponding *f*_2_ frequencies are ~ 4 to 7 kHz) so that they can be compared with SFOAEs measured in the same ear (dotted red lines). SFOAEs were elicited with a logarithmically swept tone presented at 36 dB FPL and extracted by scaling and subtracting the response to the same stimulus presented at 56 dB SPL. SFOAE amplitudes are shifted down by 10 dB to match the overall level of the reflection component, which has a similar spectral structure and phase gradient (thin dashed segments indicate responses with amplitudes less than 6 dB above the noise floor). In contrast, the amplitude and phase of the distortion component both vary slowly with frequency. Methods are described in [58]

DPOAE microstructure was attributed to two sources with different generation locations and phase behaviors shortly after the discovery of OAEs [59, 60], though a formal distinction between the underlying source mechanisms was provided later [7]. Various techniques have been used to identify the contribution of an SFOAE-like component to the DPOAE (e.g., [60, 61]), thus confirming the two-source framework. Importantly, suppressing or otherwise removing the reflection component reduces or eliminates DPOAE microstructure without necessarily reducing the overall DPOAE amplitude [62], demonstrating that microstructure results from interference by the reflection component. As an example, Fig. 8 shows the amplitudes and phases of a portion of the total DPOAE response previously shown in Fig. 7, along with the distortion and reflection components, which were separated via time-domain windowing [58]. The reflection component strongly resembles SFOAEs measured independently in the same ear.

Note that DPOAEs other than 2*f*_1_-*f*_2_ also theoretically contain various mixtures of distortion and reflection components. However, their locations of origin and behaviors may be more complex when the DP frequency is higher than *f*_2_. For instance, the 2*f*_2_-*f*_1_ DPOAE is thought to be dominated by a reflection component that originates primarily from the location tuned to the DP frequency, which is basal to the location tuned to *f*_2_ [63, 64].

### Relationships Between DPOAE Microstructure and Other Microstructures

Because the phase of the distortion component varies little with frequency, the periodicity of DPOAE microstructure is primarily determined by the phase gradient of the reflection component. The periodicity is therefore similar to that of the microstructure that arises from multiple intracochlear reflections (e.g., [65–67]). However, the actual microstructure patterns are not identical [39], as DPOAE microstructure depends on the level and phase of the reflection component relative to those of the distortion component. Complexity in DPOAE microstructure morphology is introduced by variations in the level and phase of the distortion component as well as the influence of multiple intracochlear reflections [68].

### Dependence on Cochlear Amplification

DPOAE microstructure is sensitive to cochlear status, though not always in a straightforward manner. Less microstructure is generally observed in ears with poorer hearing thresholds [69]. DPOAE microstructure is reduced and shifted to lower frequencies after noise exposure [70] and aspirin ingestion [71]. MOC activation also tends to reduce microstructure depth but shifts the microstructure pattern upward in frequency [72, 73]. These distinct effects of noise exposure versus MOC activation are similar to those previously described for the microstructure in thresholds and reflection-source emissions. Interestingly, aging in normal-hearing individuals reduces DPOAE amplitudes but not microstructure, which can actually become more prevalent in older adults [66].

The aforementioned effects on DPOAE microstructure are sometimes difficult to interpret, as they can be driven by changes in the amplitude and/or phase of one or both of the DPOAE components. Additionally, since the magnitude of the reflection component depends on the strength of the distortion-generating mechanism, changes in the distortion and reflection components are intertwined. The relative amplitude of the reflection component is also highly variable across ears and depends on the stimulus levels and frequency ratio [74, 75].

Fortunately, separating the distortion and reflection components (as shown in Fig. 8 and described below) has clarified how different manipulations or cochlear factors impact DPOAE microstructure. For instance, MOC-induced shifts in DPOAE microstructure have been shown to result from a phase lead in the reflection component, with the distortion component phase being relatively unaffected [72, 73]. In older adults, the preservation (or enhancement) of microstructure despite overall age-related declines in DPOAE amplitudes results from a larger drop in the distortion component compared to the reflection component [66]. By becoming more similar in amplitude, the two components can produce even stronger interference patterns.

### Separating DPOAE Components

Separation of the distortion and reflection components is now routine and can be achieved through a variety of suppression or time-domain techniques (reviewed in [61, 76]). Long and colleagues advanced a now widely adopted technique in which DPOAEs are measured with continuously swept stimuli and analyzed with a least-squares-fit approach [77]. By appropriately selecting the length of the analysis window applied to each portion of the response waveform, the contribution of the reflection component can be effectively filtered out, leaving just the distortion component.

As described above, DPOAE component separation provides better access to the basic properties and vulnerabilities of the distortion and reflection mechanisms. Additionally, examination of the distortion component alone reduces the significant inter- and intra-subject variability that is caused by microstructure [78]. Nevertheless, while theoretically appealing, evidence for the diagnostic utility of DPOAE component separation has so far been mixed [79, 80].

### Interference Between Short- and Long-Latency Components in TEOAEs and SFOAEs

In the context of DPOAE measurements, short- and long-latency components are typically synonymous with the distortion and reflection components. However, short-latency components have also been found to contribute to TEOAEs and SFOAEs measured at moderate-to-high stimulus levels [81, 82]. While intermodulation distortion could contribute to TEOAEs [83], it is unclear if short-latency components necessarily arise from the same mechanism that generates the DPOAE distortion component. Short-latency components may arise from nonlinear reflection [84] or reflections from more basal locations [82, 85]. Regardless of their mechanistic origins, interference between short- and long-latency components may be a general source of microstructure (and/or macrostructure) in all evoked OAEs. As discussed below, the contribution of short-latency components to SFOAE and TEOAE microstructure may differ among species.

## Comparing Microstructure Across Species

Distortion- and reflection-source emissions (including SOAEs) are measurable in many non-human mammals [86]. In common laboratory rodents, however, microstructure due to interference caused by the reflection mechanism is generally less prominent than in humans and other primates. SOAEs are also not commonly observed in rodents. In fact, rather than being a sign of a healthy cochlea, large SOAEs in rodents can appear after acoustic trauma [33] or genetic mutations that alter tectorial membrane properties [87]. The appearance of SOAEs (and other reflection-source emissions) in these disordered states could be attributed to increased irregularity in the mechanics, resulting in strong reflection as long as amplification remains sufficiently high.

Microstructure in SFOAE and TEOAE responses has not been thoroughly examined in non-human ears, perhaps because it is not a striking feature of the responses. While measurements have typically not been made at the low stimulus levels where the influence of multiple intracochlear reflections is strongest, time-domain analyses of SFOAEs and TEOAEs in guinea pigs and gerbils suggest that the contribution of these reflections is quite small [88, 89]. Interestingly, the spectral structure in the responses can actually become more pronounced at higher stimulus levels, owing to the increasing contribution of a short-latency, distortion-like component [88, 89]. As discussed in the previous section, the origins of such components remain to be fully understood, though they clearly contribute to the spectral structure of OAEs.

While DPOAE measurements in animal models are routine, there have nevertheless been few explicit studies of DPOAE microstructure. Long and colleagues characterized DPOAE microstructure in chinchillas from a colony in which SOAE prevalence was high and found that the microstructure in spontaneously-emitting ears could be as strong as in humans [90]. However, the overall impression from these and other measurements in small mammals is that DPOAE microstructure is weaker and more broadly spaced than in humans [91, 92]. In guinea pigs, for instance, DPOAEs are dominated by the distortion component under most conditions, though a reflection component with a steep phase gradient has been identified [91]. In rats, chinchillas, and rabbits, the identification of such components has been more elusive [92]. Interestingly, a form of spectral rippling observed in guinea pig DPOAEs at high frequencies is essentially unaffected by attempts to suppress the reflection component [93].

**Fig. 9:**
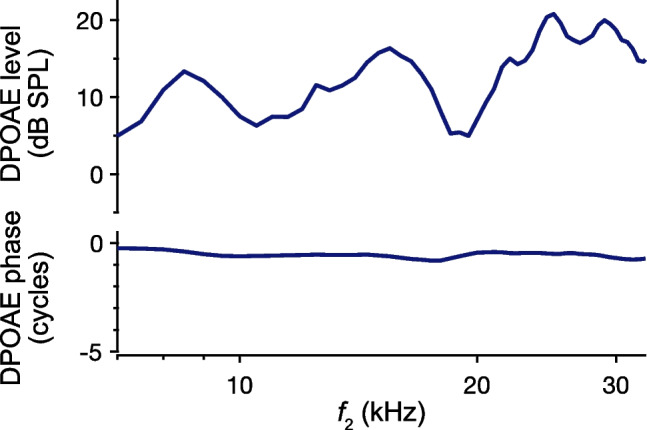
DPOAEs in mice exhibit relatively weak microstructure. DPOAE amplitudes and phases are shown as a function of the *f*_2_ frequency for a wild-type CBA/CaJ mouse. DPOAEs were measured using discrete-tone stimuli presented at 50 dB SPL, with *f*_2_ varied in 0.5 kHz steps and an *f*_2_/*f*_1_ ratio of 1.22. While there are broad amplitude peaks and valleys, the responses lack the strong, rapid variations seen in humans. Methods are described in [95]

DPOAEs in mice are similar to those in other rodents and vary more smoothly than in humans [94]. Figure 9 shows DPOAEs from an individual CBA/CaJ mouse, revealing broad peaks and valleys in the responses, with smaller, rapid fluctuations observed at higher frequencies. However, the responses clearly lack the strong microstructure that can be seen in human DPOAEs (e.g., Figs. 7 and 8). It is also uncertain if the spectral features that are observed are all of cochlear origin or if they may instead be attributed to properties of the middle ear or ear canal.

The relatively low incidence of SOAEs and weak evoked-OAE microstructure in small mammals may be a consequence of the broader mechanical tuning in these species [96]. For instance, sharp mechanical tuning may be necessary to produce large intracochlear reflections at low stimulus levels. As sharper tuning is associated with steeper traveling-wave phase gradients, broader tuning may allow for greater in-phase summation of the distortion components contributing to the total DPOAE. This could explain why DPOAEs are large and strongly dominated by the distortion component in certain species. Since the phase gradients of reflection-source OAEs are indeed shallower in laboratory animals than in humans, consistent with broader tuning [96], any interference caused by the reflection mechanism would produce more widely spaced microstructure, as is observed.

In laboratory animals, OAEs can also be elicited by electrical current applied inside of the cochlea or to the round window membrane, thus directly driving OHC activity closest to the stimulating electrodes [97, 98]. These electrically evoked OAEs (EEOAEs) can exhibit strong microstructure, particularly when the electrode is placed at the round window. EEOAE microstructure is physiologically vulnerable and appears to result from interference between short- and long-latency components, much like DPOAEs [99]. Specifically, it has been suggested that a relatively invulnerable, short-latency component is generated by OHCs near the electrode, while a more labile, long-latency component results from reflections of forward-traveling waves initiated by the electrically driven OHC activity [98, 99]. However, this idea is contradicted by the long-delay component’s frequency-independent latency, as well as its presence in mutant mice that lack traveling wave amplification, and, presumably, strong reflection-source OAEs [100]. The mechanisms underlying EEOAE microstructure and their relation to the mechanisms responsible for the microstructure in acoustically evoked OAEs remain to be elucidated.

While beyond the scope of the present review, spontaneous and evoked OAEs (including EEOAEs) are produced in a variety of non-mammalian species with diverse inner ear anatomies, including those that do not appear to support traveling waves [101–103]. Measurements from many of these species reveal some form of micro- or macrostructure. Even if the details of the generation mechanisms may be different, it is possible that similar principles apply to these ears, resulting in interference between OAE sources and resonance-like phenomena [102].

## Microstructure in Invasive Measures of Cochlear Function

Though intracochlear reflections may be relatively weak in laboratory animals, certain invasive cochlear measurements have revealed spectral ripples that may arise from the reflection mechanism. For instance, basilar membrane (BM) displacements in the chinchilla cochlea sometimes exhibit small, quasiperiodic variations with frequency when elicited with low-level tones [104, 105]. BM displacement phases accumulate ~ 0.5 cycles between adjacent displacement maxima, consistent with the ripples being due to reflection back and forth between a location near the measurement site and the middle-ear boundary. The ripple spacing is also correlated with that observed in the ear-canal pressure, indicating a common dependence on the SFOAE-generating reflection mechanism.

Ripples in BM responses are not observed in all chinchillas, possibly due to suboptimal cochlear sensitivity, and their general prevalence in other species is unknown. Optical coherence tomography, which does not require opening and potentially damaging the cochlea, could be useful for further examining microstructure in mechanical responses. A key advantage of this technique is that vibration measurements are not limited to single cochlear locations in each preparation [95]. Thus, rather than attempting to find ripples in the responses at a single location, it may be possible to identify spatial variations in sensitivity that are more analogous to what drives threshold microstructure.

Such a panoramic view of response sensitivity was previously explored in cat and chinchilla, albeit by compiling responses from auditory nerve fibers with many different characteristic frequencies [106]. Spatial irregularities in spontaneous and driven firing rates were evident in responses from individual ears, though they were also somewhat preserved when pooled across animals. Since threshold and OAE microstructures are idiosyncratic to each ear, it is unclear whether the ripples in nerve fiber responses are related to multiple intracochlear reflections or if they are due to some other spatial irregularity. It will be challenging to follow up on this intriguing finding given the sheer amount of data that was required to generate it.

Most recently, microstructure has been identified in the cochlear microphonic recorded from the round window in chinchilla [107]. The cochlear microphonic originates from the summation of OHC transduction currents, with the largest contribution coming from OHCs at the basal end of the cochlea. However, analysis and modeling of spectral ripples observed in the microphonic revealed interference between multiple components: short-latency components from basal OHCs, mid-latency components from the tonotopic place of the stimulus frequency, and long-latency components from basal OHCs stimulated by intracochlear reflections. Due to the mixing of three different components, the microstructure in the microphonic may be somewhat more complex than that observed in the SFOAEs elicited by the stimulus tones. Regardless, these recent findings highlight the possibility that there may be spectral structures that have yet to be discovered in various cochlear responses.

## Conclusions

Many psychoacoustic and OAE measurements reveal quasiperiodic fluctuations in frequency that are idiosyncratic to a given ear. These microstructure patterns are currently understood to originate from intracochlear wave interference that is initiated by the mechanisms underlying reflection-source emissions. While fascinating as auditory “fingerprints,” microstructure patterns also seriously complicate inter- and intra-subject comparisons of both threshold and supra-threshold responses. Fortunately, owing to the detailed measurements of Glenis Long and colleagues, as well as many others along the way, we have come to better understand the mechanisms underlying microstructure and developed techniques to reduce its influence. Nevertheless, further work is needed to refine and test the diagnostic utility of these techniques. Additionally, more systematic characterization of the micro- and macrostructures in distortion and reflection emissions across species would be useful for clarifying potential species differences in the underlying mechanisms. Such measurements may identify alternative sources of spectral structure in cochlear responses that have yet to be appreciated.

## Data Availability

All data plotted in this review are available at https://doi.org/10.6084/m9.figshare.27964554.

## Code Availability

All code used to generate figures is also available at https://doi.org/10.6084/m9.figshare.27964554.
